# AI/ML‐Driven DPP‐4 Inhibitor Predictor (d4p_v1) for Enhanced Type 2 Diabetes Mellitus Management: Insights Into Chemical Space, Fingerprints, and Electrostatic Potential Maps

**DOI:** 10.1002/ardp.70106

**Published:** 2025-09-22

**Authors:** Anu Manhas, Ritam Dutta, Stefano Piotto, Sk. Abdul Amin

**Affiliations:** ^1^ Department of Chemistry, School of Energy Technology Pandit Deendayal Energy University Gandhinagar India; ^2^ Department of Pharmaceutical Technology JIS University Kolkata West Bengal India; ^3^ Department of Pharmacy University of Salerno Fisciano SA Italy

**Keywords:** chemical space, DPP‐4, electrostatic potential, machine learning, scaffold analysis

## Abstract

Dipeptidyl peptidase‐4 inhibitors (DPP‐4i) represent a relatively new class of oral antidiabetic drugs. This study focuses on: (a) identifying favourable and unfavourable fingerprints governing DPP‐4 inhibition using fragment‐based analysis, (b) validating key fingerprints through HOMO–LUMO gap analysis and electrostatic potential (ESP) maps, and (c) developing AI/ML‐driven DPP‐4 predictor, an online cheminformatics tool for efficient DPP‐4i screening using a trained, validated AI/ML model. The fragment‐based QSAR model finds key substructures linked to potent DPP‐4 inhibition, including 2‐cyanopyrrolidine, 3‐amino tetrahydropyran, and difluoro phenyl groups. D0010 (3‐aminotetrahydropyran fingerprint G10) is the most reactive, while D0094 (difluorophenyl fingerprint G14) is the most stable, with D0012 and D0013 (2‐cyanopyrrolidine fingerprints G1, G5) offering a balance between stability and reactivity. In addition, the d4p_v1 tool (https://github.com/Amincheminfom/d4p_v1) reliably distinguishes active and inactive DPP‐4i using molecular descriptors derived from input SMILES strings. Therefore, this study not only revealed the chemical space of DPP‐4i but also opened up a horizon in developing novel potent DPP‐4i for the management of type 2 diabetes mellitus (T2DM) in the future.

## Graphical abstract

Favourable and unfavourable fingerprints governing dipeptidyl peptidase‐4 (DPP‐4) inhibition are identified using fragment‐based analysis, validating key fingerprints through HOMO–LUMO gap analysis and electrostatic potential maps. AI/ML‐driven DPP‐4 predictor, an online cheminformatics tool for efficient DPP‐4 inhibitor screening, is reported.

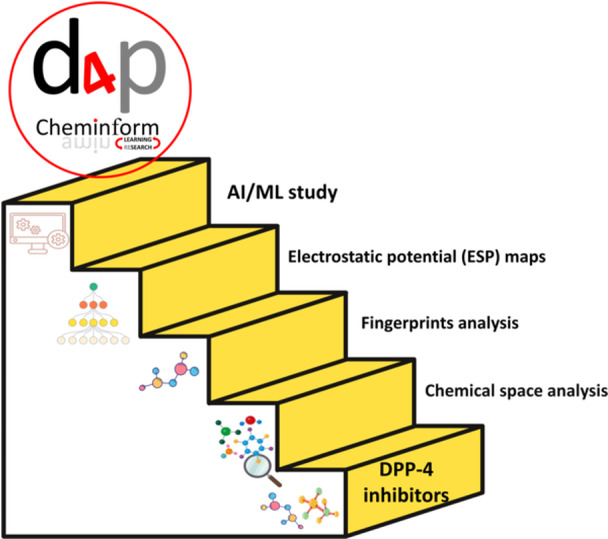

## 1 Introduction

Type 2 diabetes mellitus (T2DM) has become a public health emergency of the 21st century, affecting hundreds of millions of people across the world and creating tremendous socioeconomic and healthcare challenges [1]. It is estimated that more than 537 million adults worldwide have diabetes, with type 2 diabetes being responsible for the overwhelming majority of these numbers, and the rates of prevalence continue to escalate alarmingly. For decades, the foundation of T2DM pharmacotherapy was based on drugs designed to enhance insulin secretion or suppress hepatic glucose production. Although useful at reducing blood glucose, earlier drug classes such as sulfonylureas and thiazolidinediones are frequently linked to major side effects. These restrictions underlined the urgent need for new therapeutic modalities that were able to efficiently control hyperglycemia with better safety profiles and fewer adverse side effects.

**Figure 1:**
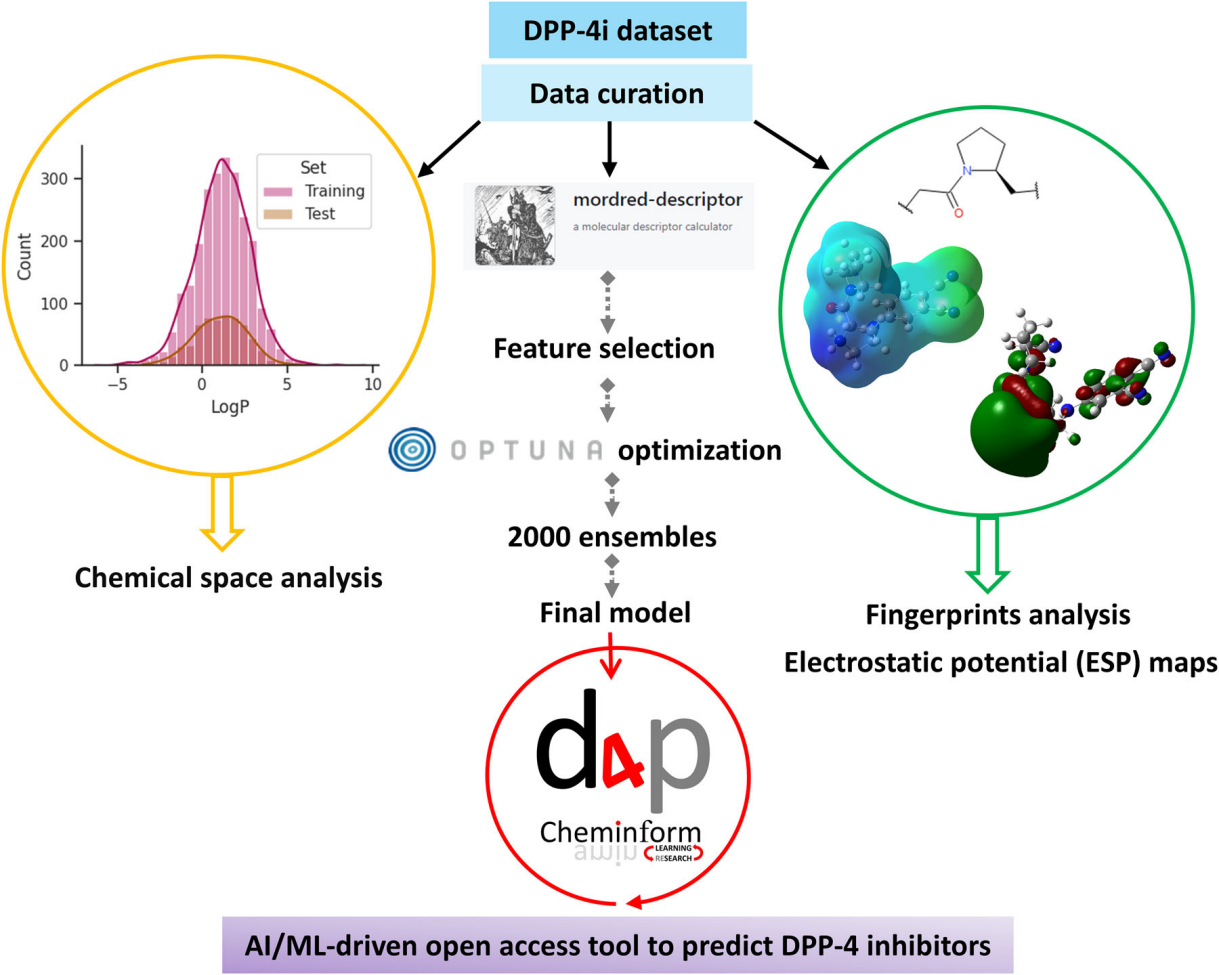
An overview of the study design. This study is an initiative to explore the structure requirements of DPP‐4 inhibitors.

Dipeptidyl peptidase‐4 inhibitors (DPP‐4i) have surfaced as a worthy and comparatively younger category of oral antidiabetic drugs [2, 3, 4, 5, 6]. These drugs work based on an unconventional mechanism related to the incretin hormone mechanism, a natural mechanism in the gastrointestinal tract that has an essential function in maintaining glucose homeostasis. DPP‐4 is an ubiquitous serine protease enzyme that spontaneously cleaves incretin hormones, including mainly glucagon‐like peptide‐1 (GLP‐1) and glucose‐dependent insulinotropic polypeptide (GIP). The incretins are secreted from the intestine upon food intake and have profound glucose‐lowering activities. They accomplish this by increasing insulin release from pancreatic beta cells in a glucose‐dependent manner, that is, release is increased when blood glucose is high and decreased when it is low, thus minimizing the risk of hypoglycemia [7, 8, 9, 10, 11]. At the same time, incretins inhibit glucagon secretion, a hormone that opposes insulin by increasing blood glucose, which adds to enhanced glycemic control. Hence, DPP‐4i is a useful weapon in the therapeutic arsenal for the treatment of T2DM, either as monotherapy or in combination with other antidiabetic drugs [12]. This study has three principal focuses: (i) a step to explore the favourable and unfavourable fingerprints (sub‐structural fragments) that regulate DPP‐4 inhibition, using fragment‐based quantitative structure–activity relationship (QSAR) modelling, (ii) confirmation of the significance of identified fingerprints through highest occupied molecular orbital (HOMO) and lowest occupied molecular orbital (LUMO) gap of ligand and exploration of electrostatic potential (ESP) maps, and (iii) the development of an innovative online cheminformatics tool that enables researchers to efficiently screen potential DPP‐4i using a trained and thoroughly validated artificial intellengecne/machine learning (AI/ML) model (Figure 1).

## 2 Result and Discussion

### 2.1 Fingerprint‐Based Study

#### 2.1.1 Chemical Space Analysis

Chemical space plays a crucial role in chemical and biological research, especially in pharmaceutical chemistry. In our study, we used a data‐driven approach to identify chemical motifs and explore their structure–activity relationship (SAR) implications. A systematic analysis of the Structure‐*Simila*rity *ACt*ivity *Trail*ing (SimilACTrail) map (https://similactrailv1web.streamlit.app) for DPP‐4i based on Tanimoto Similarity (*Tc*) [13, 14] provides valuable insights into the activity landscape of these compounds. Notably, *activity cliffs* [15] account for just 0.02% of all pairs.

#### 2.1.2 Scaffold Content and Diversity Analyses

**Figure 2:**
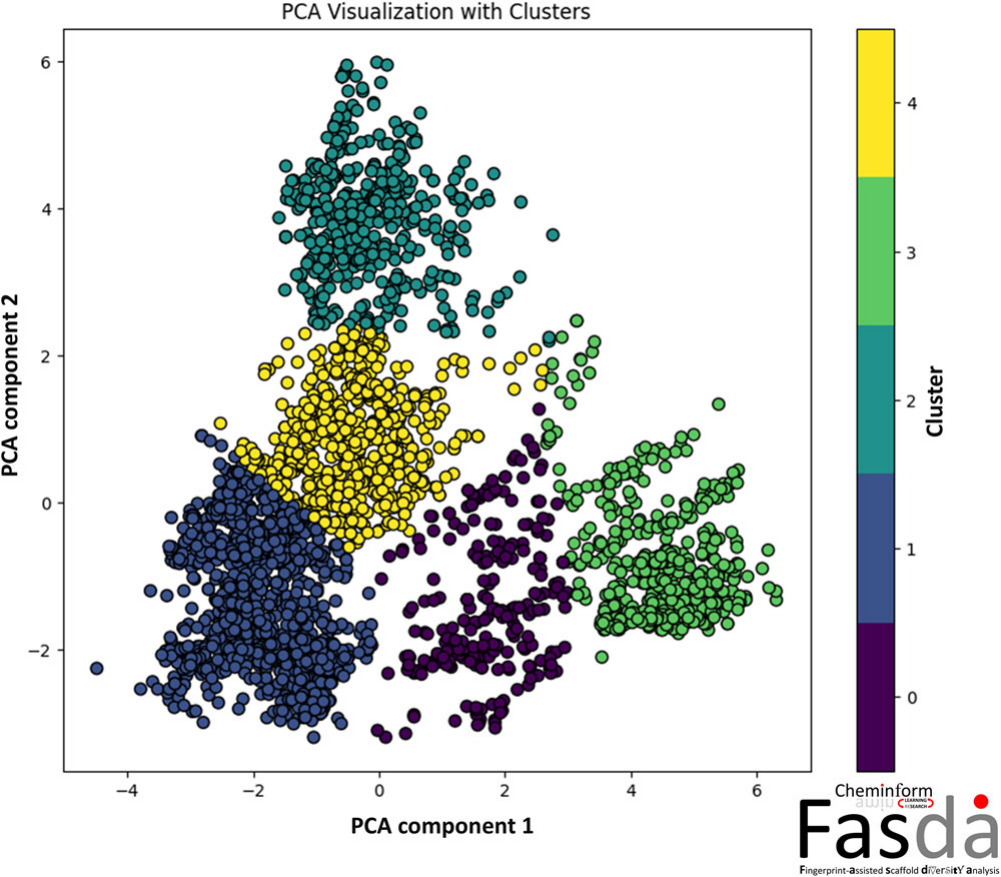
The PCA scatter plot highlights the molecules from each cluster of DPP‐4i. This plot is generated from the freely available Fasda_v1.0 tool (https://fasdav1web.streamlit.app/).

The scaffold content and diversity calculation of DPP‐4 inhibitors were performed by Fasda_v1.0 tool (https://fasdav1web.streamlit.app/) [16]. After generating the Extended Connectivity Fingerprint (*ECFP4*), a pairwise similarity matrix was calculated using the Tanimoto similarity metric. Principal component analysis (PCA) was then applied to reduce the high‐dimensional fingerprint data into a 2D space for visualization. Subsequently, k‐means clustering analysis (k‐MCA) was performed on the PCA‐transformed data, resulting in five clusters. This Fasda_v1.0 tool also analyzed the number of compounds (M), number of Bemis–Murcko (unique) scaffolds (N) [17], number of singleton scaffolds (Ns), the singleton ratio (Ns/N). Typically, “Bemis‐Murcko scaffold” [17] represents the main ring systems and linker atoms, whereas the “singleton scaffolds” [18] refer to molecular scaffolds that are only observed once in a data set or cluster. The results are provided in Table 1. The PCA scatter plot highlighting the molecules from each cluster of DPP‐4i is depicted in Figure 2.

**Table 1:** Diversity calculation of DPP‐4i data set.

Cluster	Number of compounds	Unique scaffolds	Singleton scaffolds	Singleton ratio
**0**	259	132	96	0.727
**1**	1224	511	340	0.665
**2**	449	183	119	0.650
**3**	489	182	118	0.648
**4**	640	293	208	0.710

As observed from Table 1, Cluster 0 contains 259 compounds. Cluster 0 is also represented by 132 distinct Bemis–Murcko scaffolds and has a singleton ratio of 0.727. Cluster 1 is the largest cluster among all (*M* = 1224). It is represented by 511 distinct Bemis–Murcko scaffolds (singleton ratio = 0.665). Clusters 2 and 3 contain 449 and 489 compounds, respectively. Cluster 4 contains 640 compounds, and this cluster is represented by 293 distinct Bemis–Murcko scaffolds (singleton ratio = 0.710). In comparison, Cluster 0 (0.727) and Cluster 4 (0.710) exhibit higher singleton ratios, suggesting that their scaffolds are shared across a larger proportion of compounds. Overall, the diversity analysis and scaffold breakdown indicate that all clusters in this data set contain more than 64% singleton ratios. This emphasizes the diversity present across the data set.

#### 2.1.3 Data Set Division

DPP‐4 inhibitors with *pIC*_*50*_ <= 7.03 were assigned as “inactives” (0 = 1531), and compounds with *pIC*_*50*_ > 7.03 were assigned as “actives” (1 = 1530). The *k*‐means clustering analysis (*k‐*MCA) [19] was performed with descriptors including molecular weight (*MW*), the number of hydrogen bond donors (*nHBD*), the number of hydrogen bond acceptors (*nHBA*), the number of rotatable bonds (*nRB*), the number of aromatic rings (*nAR*), and lipophilicity (*LogP*) to divide the data set (*N*_Total_ = 3061) into training and test sets.

**Figure 3:**
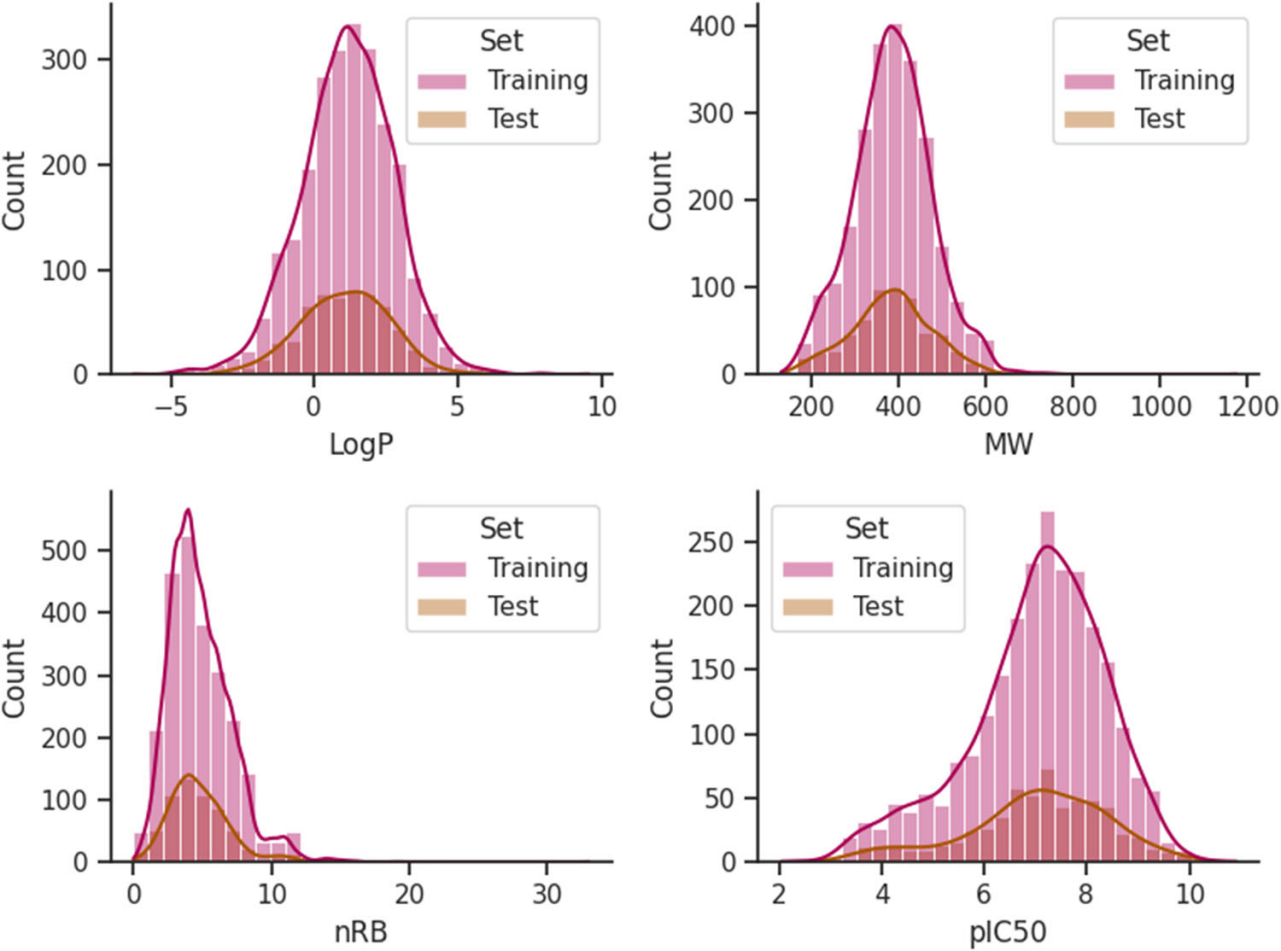
Bin plots of *LogP*, *MW*, *nRB*, and *pIC*_*50*_ features.

From each cluster, 20% of test set compounds (*N*_Test_) were selected, and the remaining 80% of molecules were considered as training set compounds (*N*_Train_ = 2448). Further, the bin plots with histograms of features *LogP*, *MW*, *nRB*, and *pIC*_*50*_ (Figure 3) have been drawn to visualize the chemical distribution of the compounds. This plot also helps to understand the distribution of the test set compounds truly represents the training set.

#### 2.1.4 Bayesian Classification Study

The Bayesian classification model was built using various properties, including the extended connectivity fingerprint at diameter 6 (*ECFP6*). The model yielded an *ROC*_*LOO*_ of 0.872. Additionally, fivefold cross‐validation resulted in an *ROC*_*5CV*_ of 0.854 for the training set, and the test set achieved an *ROC*_Test_ of 0.828. The performance of the developed Bayesian classification model was further evaluated using several metrics: sensitivity (*Se*) of 0.880, concordance (*Conc*) of 0.873, *F1*‐score of 0.894, Matthews correlation coefficient (*MCC*) of 0.737, and Cohen's Kappa (*k*) of 0.736 for the training set. In addition, the test set exhibits a *Se* of 0.788, *Conc* of 0.725, *F1*‐score of 0.708, and as depicted in Table 2.

**Table 2:** Validation metrics of the developed Bayesian model of DPP‐4 inhibitors.

Set	*TP*	*FN*	*FP*	*TN*	*Se*	*Conc*	*ROC*	*F1*
Train^a^	1307	179	132	831	0.880	0.873	0.854	0.894
Test	204	55	113	240	0.788	0.725	0.828	0.708

^a^ Fivefold cross‐validation; sensitivity (*Se*), concordance (*Conc*).

#### 2.1.5 Bayesian Classification Model Generated Fingerprints Analysis

**Figure 4:**
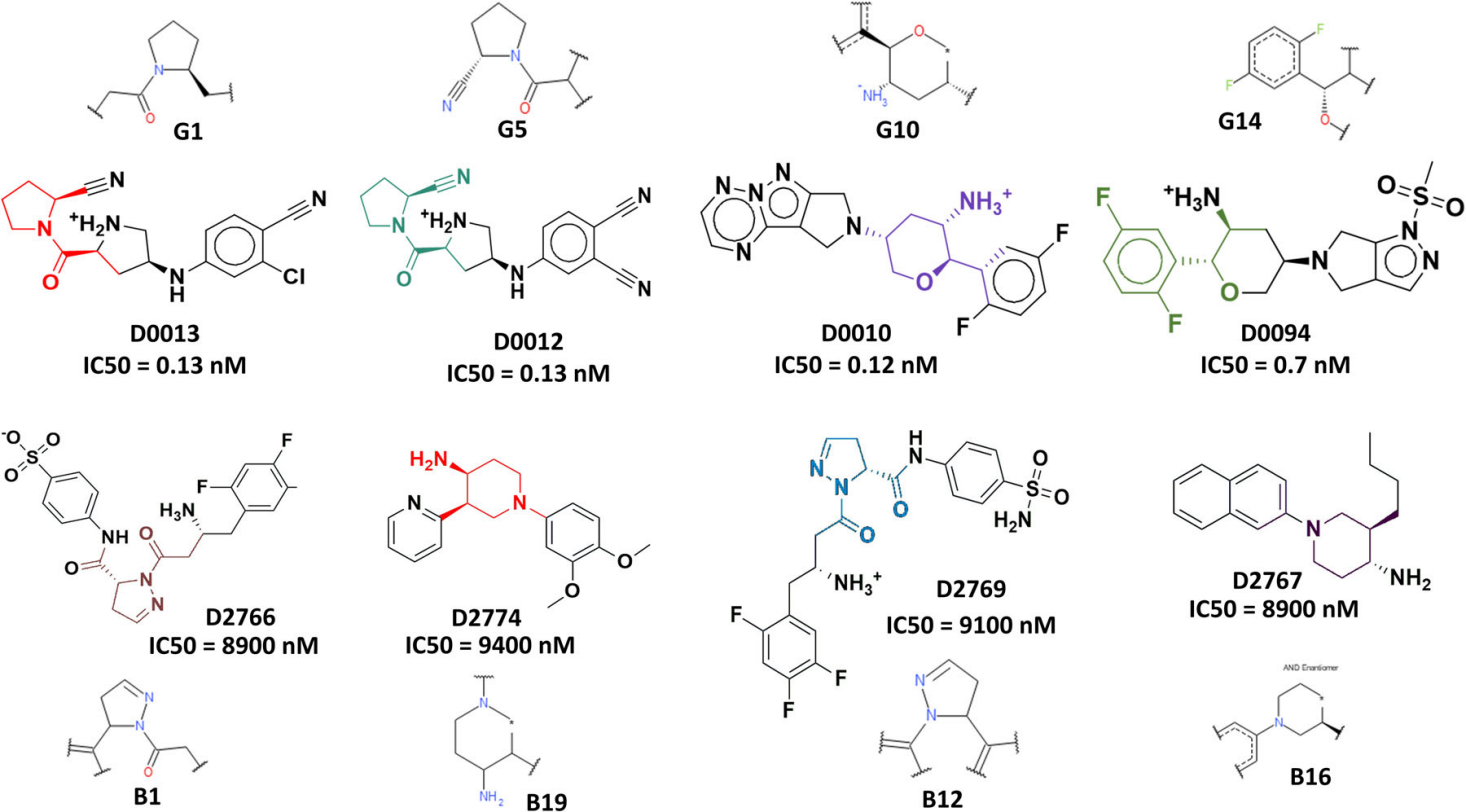
Bayesian fingerprints positively (G1, G5, G10, and G14) and negatively (B1, B19, B12, and B16) influence DPP‐4 inhibitory activity along with the corresponding inhibitors.

Bayesian classification identified 20 good (G1‐G20) and 20 bad (B1‐B20) fragments that enhanced and diminished DPP‐4 inhibitory activity, respectively. These 20 good fragments (G1‐G20) can be classified into three major groups, namely, 2‐cyanopyrrolidine, 3‐amino tetrahydropyran, and difluoro phenyl groups. Likewise, the bad fragments (B1‐B20) can be classified into two classes such as pyrazolines with β‐amino acyl function, 4‐aminopiperidines. Compounds D0012 and D0013 having 2‐cyanopyrrolidine group (i.e., G1, G5 fingerprints, Figure 4) exhibit excellent DPP‐4 inhibitory activity [20].

Notably, pyrrolidine derivatives have been extensively investigated as DPP‐4 inhibitors due to the specificity of the enzyme for substrates featuring an amino‐terminal proline at the C2 position. These derivatives mimic the cleavage product of the P2–P1 dipeptidyl substrate, where the P1 site incorporates a proline‐like structural motif. Consequently, 1‐(γ‐substituted prolyl)‐(S)‐2‐cyanopyrrolidines not only demonstrate promising nanomolar inhibition of DPP‐4 by acting as proline mimics but also exhibit sufficient chemical stability for oral administration [12]. Additionally, the presence of 3‐amino tetrahydropyran function in a compound influences the DPP‐4 inhibitory activity (e.g., D0010: DPP‐4 IC_50_ = 0.12 nM, Figure 4). The importance of difluoro phenyl moiety is suggested by the substructural features G14. This observation can be analyzed by D0094 containing difluoro phenyl moiety, exhibiting promising activity (DPP‐4 IC_50_ = 0.7 nM). In contary, the negative influence of pyrazoline function with β‐amino acyl moiety is suggested by the substructural features B1, B12. This observation can be explained by D2766 (DPP‐4 IC_50_ = 8900 nM) and D2769 (DPP‐4 IC_50_ = 9100 nM), both of these DPP‐4i are poorly active. Substructural features B16 and B19 show the influence of 4‐aminopiperidines function toward the poor DPP‐4 inhibitory activities. For instance, D2774 and D2767 containing bad fragments B19 and B16, respectively, show poor activity (D2774: DPP‐4 IC_50_ = 9400 nM and D2767: DPP‐4 IC_50_ = 8900 nM, Figure 4).

In our previous study, analysis of 104 DPP‐4 inhibitors highlighted the importance of certain structural features [21], specifically, the adamantyl ring and N‐substituted‐2‐cyano‐pyrrolidides were identified as favorable for enhancing DPP‐4 inhibitory activity. Meanwhile, the comparison between the previous [21] and current findings underscores the consistent importance of 2‐cyanopyrrolidine moieties in enhancing DPP‐4 inhibitory activity, while also identifying additional favorable and unfavorable features that provide a more comprehensive understanding of the SAR for DPP‐4i.

### 2.2 Confirmation of the Significance of Identified Fingerprints

#### 2.2.1 Frontier Molecular Orbital Analysis

**Figure 5:**
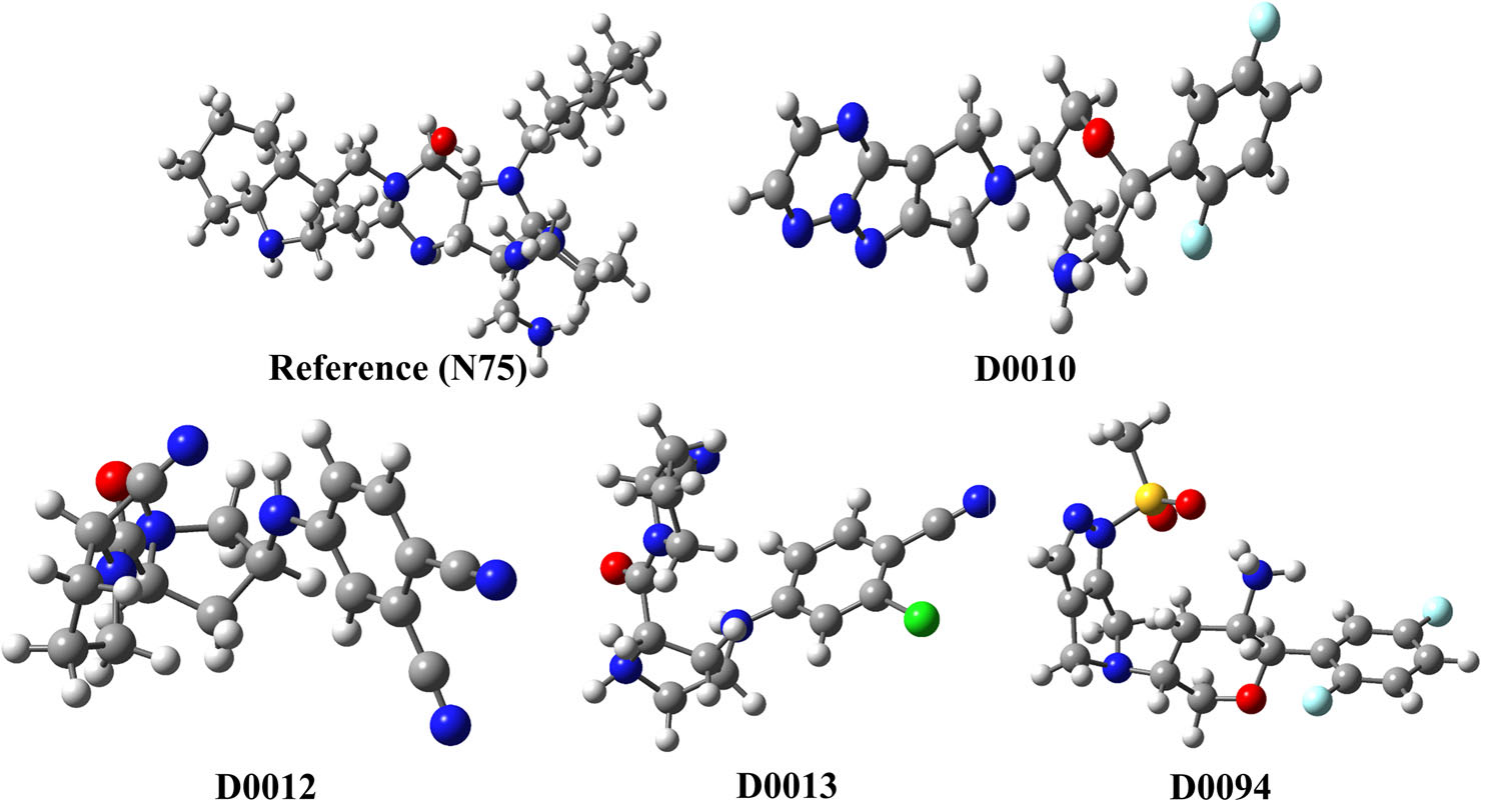
Optimized geometries of the compounds retrieved at B3LYP/6‐31+g(d) level of theory.

**Figure 6:**
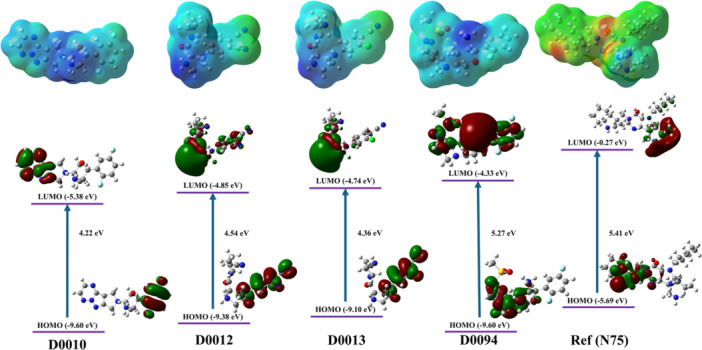
Pictorial representation of HOMO–LUMO energy gap, molecular orbitals, and electrostatic potential maps of the selected compounds obtained at B3LYP/6‐31+g(d) level of theory.

The ground state optimized geometries of the selected compounds D0010, D0012, D0013, D0094, and reference (N75) are shown in Figure 5. The frontier molecular orbitals of the studied molecules are shown in Figure 6. The HOMO and LUMO, and their properties, help in the analysis of the chemical reactivity of the molecules.

The HOMO and LUMO orbital possess the tendency to donate and accept the electrons, respectively, during the molecular interaction. Thus, HOMO energy corresponds to ionization potential, and LUMO energy corresponds to electron affinity [22]. Table 3 provides insights into HOMO and LUMO energy and gap within them. They represent the stability and molecular reactivity.

**Table 3:** Values of HOMO, LUMO energy, and energy gap (eV) of the selected compounds solved at B3LYP/6‐31+g(d) level of theory.

Molecules	*E*_HOMO_ (eV)	*E*_LUMO_ (eV)	Energy gap (eV)
D0010	–9.60	–5.38	4.22
D0012	–9.38	–4.85	4.54
D0013	–9.10	–4.74	4.36
D0094	–9.60	–4.33	5.27
Reference (N75)	–5.69	–0.27	5.41

High energy gap represents stability and less chemical reactivity, as it is energetically not favourable to add an electron in the LUMO [23]. Whereas a smaller energy gap represents high chemical reactivity of the molecule [23]. It is clear from Table 3 that molecule D0010 shows the lowest energy gap of 4.22 eV, followed by D0013 (4.36 eV), D0012 (4.54 eV), D0094 (5.27 eV), and the reference with the gap of 5.41 eV. In general, lower HOMO–LUMO gap indicates the high reactivity of the molecule. On comparison, D0010 has the smallest energy gap; thus, it is the most reactive molecule among all, followed by D0013, D0012, D0094, and Reference in the order of the chemical reactivity. It can be observed that the presence of substituents increases the reactivity of the molecules. The calculated HOMO and LUMO energies are listed in Table 3. The presence of substituents effects the HOMO and LUMO energies of the studied molecules. On observing the energy of HOMO, it is clear that the molecules D0010 and D0094 have the lowest energy, thus making them less likely to donate the electrons. The presence of electron‐withdrawing substituents leads to the decrease in the energy level of the orbitals. However, molecule D0013 has the highest HOMO, making it slightly better electron donor in comparison to others. On observing the LUMO energy of the compounds, it is observed that the compound D0010 shows the lowest energy, thus making it a stronger electron acceptor. Overall, D0094 is the most stable molecule among the selected four molecules. However, molecules D0012 and D0013 possess the capability to balance within stability and reactivity, thus making them as potential candidates for displaying interactions with the proteins.

#### 2.2.2 ESP Map Analysis

The ESP analysis helps in visualizing the charge distribution over the surface of the molecule [24]. It represents the positive and negative charge showing electrophilicity and nucleophilicity, respectively. In ESP plots, red and yellow color represents the region with high electron density, and blue color represents the region with low electron density, respectively [25]. From the ESP plots shown in Figure 6, it seems that the shortlisted molecules have the capability to attract the nucleophilic region or nucleophilic residues of the protein [25]. Apart from the energy gap, other calculated parameters are shown in Table 4. These parameters were derived using the HOMO and LUMO energy levels. These parameters provide insight into the stability, reactivity, and electron‐transfer properties of the molecules. Electrophilicity index (ω) calculates the chemical reactivity and favorable change in energy on the addition of an electron [26].

**Table 4:** Values of reactive descriptors of the selected compounds calculated at B3LYP/6‐31+g(d) level of theory.

Molecules	Electrophilicity index (ω) (eV)	Chemical potential (µ) (eV)	Hardness (ɳ) (eV)	Softness (S) (eV)	Electronegativity (χ) (eV)
D0010	13.29	–7.49	2.11	0.47	7.49
D0012	11.18	–7.12	2.27	0.44	7.12
D0013	10.98	–6.92	2.18	0.46	6.92
D0094	9.21	–6.97	2.64	0.38	6.97
Reference (N75)	1.64	–2.98	2.71	0.37	2.98

Chemical potential (µ) represents the electron‐donating and accepting tendency of the compounds. Moreover, electronegativity (χ) calculates the electrophilicity of the compound as it describes the electron affinity [27]. Other parameters like hardness and softness elucidate the stability and reactivity. In chemical potential, a more negative value means higher reactivity, and a lower value means higher stability. It is clear from Table 4 that molecule D0010 is the most reactive compound, with higher electronegativity, softness, and electrophilicity. Additionally, it has the highest tendency of electron exchange. This property makes it a suitable candidate for forming interactions with the macromolecules. However, compound D0094 is the most stable among all four ligands, owing to its high hardness, low softness, and low electrophilicity. The remaining two compounds, D0012 and D0013, have a moderate tendency for exchanging the charge, which suggests a balance within the stability and reactivity. Collectively, these parameters help to study the reactivity, electron transfer ability, and stability of the compounds that help to understand the binding of the molecules with the protein.

### 2.3 Machine Learning (ML) Model Development

#### 2.3.1 Data Pretreatment and Feature Selection

Descriptors were calculated by using *in‐house* code written in the Python environment [28, 29]. This code initialized the Mordred calculator [30] with available 1614 descriptors (except the 3D descriptors). Feature selection is a crucial step in ML for improving model performance through effective data preprocessing. By identifying the most informative features, it becomes easier to understand the patterns and relationships within the data. In this study, the *Information Gain* (mutual information, MI) approach [31] was utilized to select descriptors with importance scores above 0.15. The *Information Gain* method assesses the relevance of each feature (descriptor) in relation to DPP‐4 inhibitory activity. From a set of 1288 descriptors, 13 were chosen based on their importance scores exceeding the 0.15 threshold. These selected features are considered to carry significant information about the target variable, providing valuable insights into the molecular descriptors that impact DPP‐4 inhibitory activity in chemical compounds [30, 32].

#### 2.3.2 Results of the Random Forest (RF) Models

The hyperparameters of the RF model [33] were optimized (Supporting Information S1: Figure S3) using *Optuna* [34]. The model tuning with a trail of 2000 times led to the selection of the following hyperparameters (Best hyperparameters: *n_estimators*: 72, *max_depth*: 29, *min_samples_split*: 8, *min_samples_leaf*: 9), which optimize the performance of the RF model. The *n_estimators* is found to be 72, this means that 72 trees are determined to be optimal (Supporting Information S1: Figure S3). A depth of 29 indicates that each tree can grow up to 29 levels. Limiting the depth helps prevent overfitting, where the model learns noise in the training data rather than generalizable patterns. The *min_samples_split* [35] is found to be 8; this means that if a node has fewer than eight samples, it will not be split further. This can help ensure that each split is meaningful and that the model remains generalized. Notably, *min_samples_leaf* of nine denotes that any leaf must contain at least nine samples (Supporting Information S1: Figure S3). The RF model demonstrates strong performance, achieving an accuracy of 88% on the training set (Table 5).

**Table 5:** Validation metrics of the developed RF model of DPP‐4 inhibitors.

Set	*Se*	*Ac*	*Pr*	*F1*	*MCC*	*k*
Train	0.890	0.880	0.873	0.881	0.760	0.760
Test	0.841	0.830	0.819	0.830	0.661	0.660

Abbreviations: *Ac*, accuracy; *k*, Cohen's Kappa; *MCC*, Matthews correlation coefficient; *Pr*, precision; *Se*, sensitivity.

**Figure 7:**
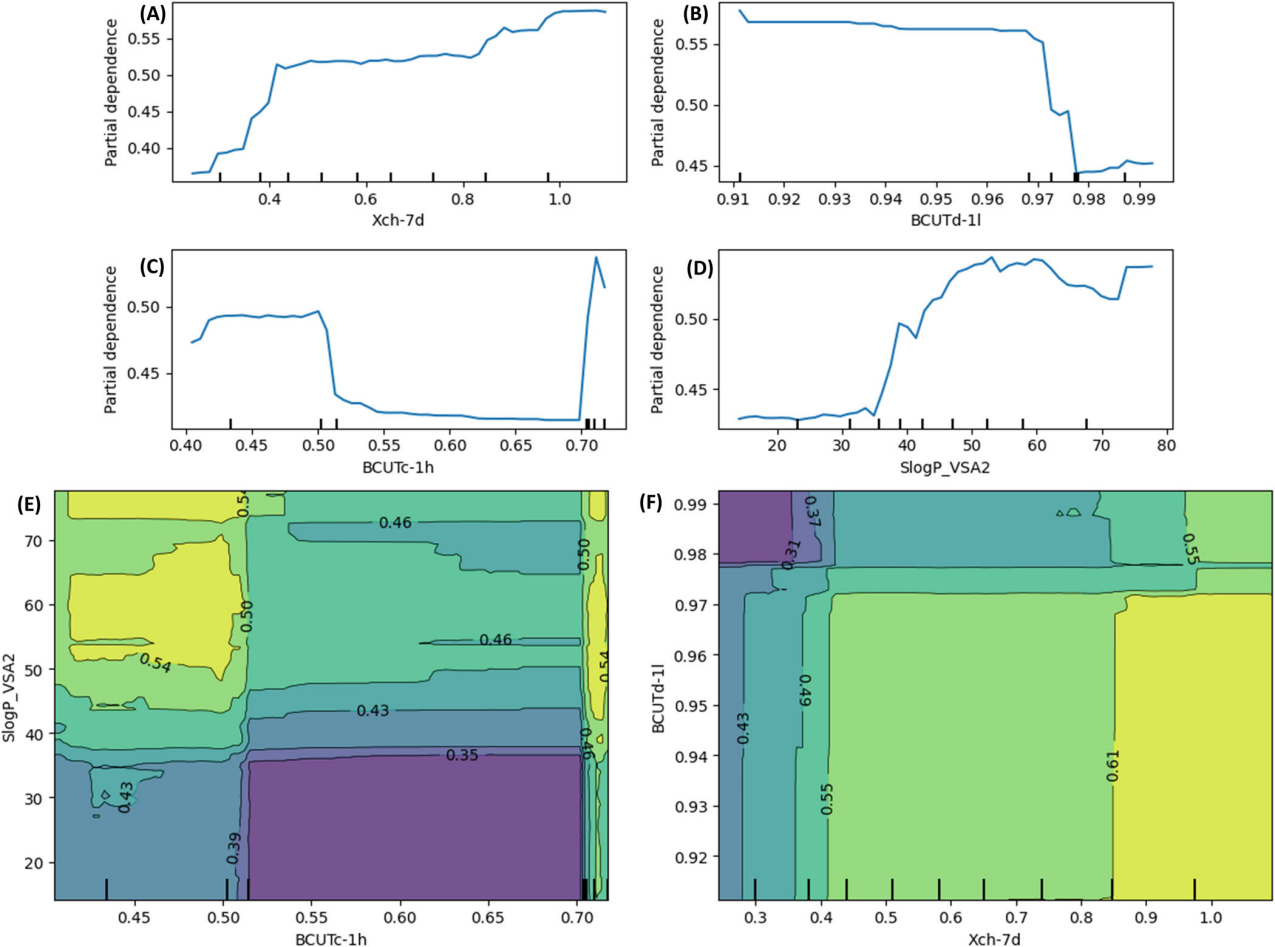
Partial dependence plot (PDP) of descriptors (A) *Xch‐7d*, (B) *BCUTd‐1l*, (C) *BCUTc‐1h,* and (D) *SlogP_VSA2*. Two‐variable PDP of chemical descriptors (E) *BCUTc‐1h versus SlogP_VSA2* and (F) *Xch‐7d versus BCUTd‐1l*.

On the test data set, the model correctly classified 83% of instances, with a precision of 81.9% further supporting its reliability. To assess model robustness, three additional ensembles of training and test sets were used to develop new models. The performance of these models was consistent with the original RF model, highlighting its stability and predictive reliability. Furthermore, the applicability domain (AD) is crucial for evaluating the uncertainty in predicting its performance [36]. In this study, the leverage approach was used to identify X‐outliers within the training set and to detect compounds falling outside the AD in the test set [number of descriptors = 9, number of training set DPP‐4i = 2448; leverage threshold: 0.011]. The analysis revealed that 598 DPP‐4i (out of 613 test set compounds) fall within the defined AD, whereas the number of compounds outside AD are 15, those exceeding the defined threshold of 0.011. Additionally, a partial dependence plot (PDP) was generated to illustrate how individual features influence the predicted outcome of the AI/ML model (Figure 7).

Two‐variable PDP of chemical descriptors demonstrate the changes in a particular feature or a set of features (i.e., *Xch‐7d*, *BCUTd‐1l*, *BCUTc‐1h*, and *SlogP_VSA2*) influence the DPP‐4 predictions while averaging out the effects of all other features. These descriptors such as *Xch‐7d*, *BCUTd‐1l*, *BCUTc‐1h*, and *SlogP_VSA2* can be indirectly related to electronic and structural features reflected in the HOMO–LUMO analysis, reinforcing the consistency between ML‐derived feature importance and quantum chemical parameters. The performance metrics of the descriptors are likely being illustrated, with the highest values concentrated in the yellow‐green regions, indicating optimal performance [37]. In contrast, the darker blue and purple areas correspond to lower values, signifying poorer performance.

#### 2.3.3 d4p_v1: An AI/ML‐Based Computational Tool to Screen DPP‐4 Inhibitors

**Figure 8:**
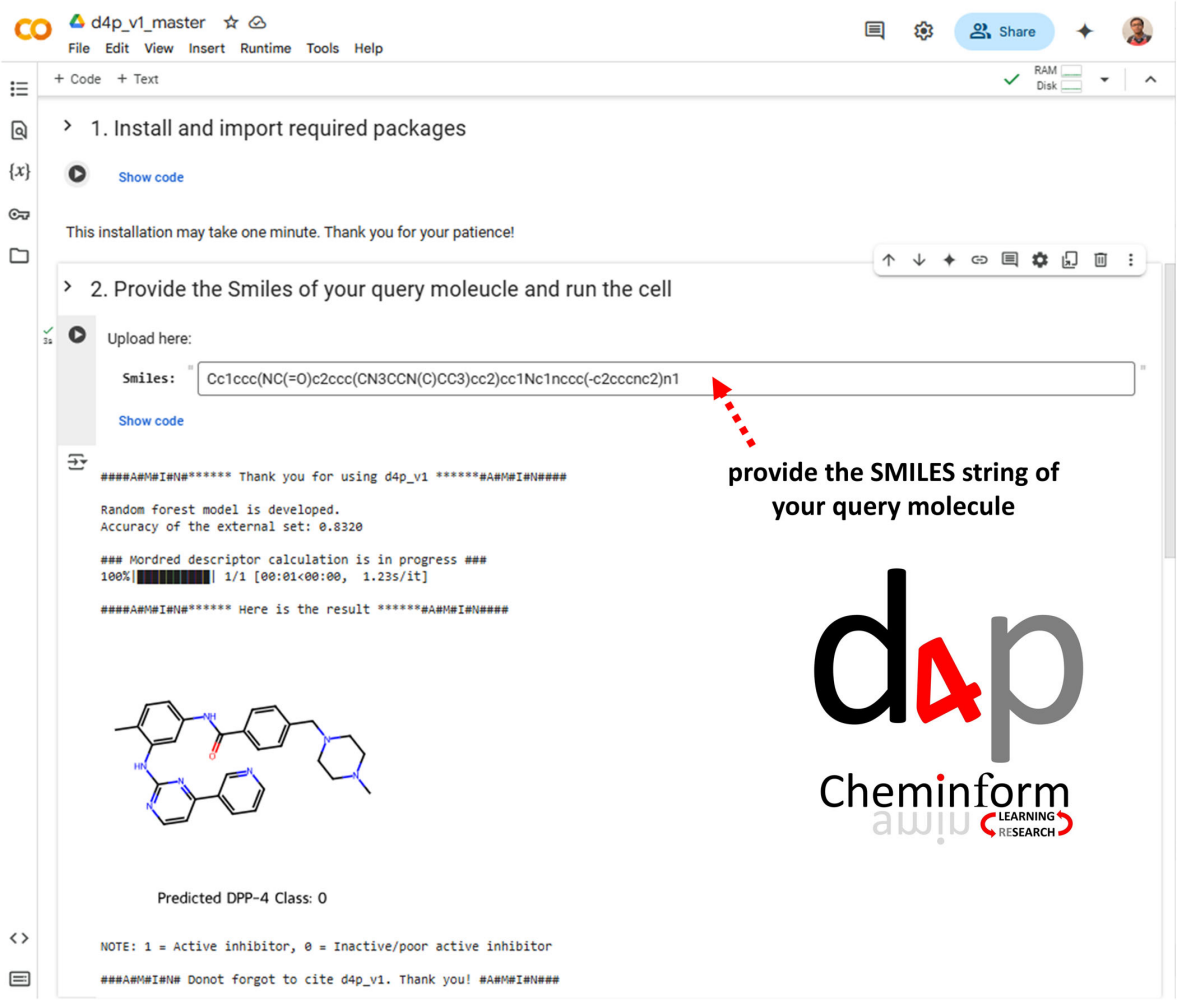
The Google colab interface of the “d4p_v1” tool. Users can predict the activity of a single compound by providing the SMILES string of the query molecule.

The “d4p_v1” is an online cheminformatics tool, accessible via Google Colab (Figure 8), that enables researchers to efficiently screen potential DPP‐4i using a pre‐trained and thoroughly validated AI/ML model. This is freely available through the link https://github.com/Amincheminfom/d4p_v1. Users can predict the activity of a single compound by providing the SMILES string of the query molecule. The tool automatically calculates the required descriptors for the input compound (query molecule), and predicts whether the query molecule is: (a) Active (Class 1): indicating potential DPP‐4 inhibitory activity, or (b) Inactive (Class 0): indicating the compound is poor active or unlikely to inhibit DPP‐4 (Figure 8).

Key elements of this tool are—(i) the model has been validated, ensuring its reliability in distinguishing between *active* and *inactive* DPP‐4i, (ii) it uses molecular descriptors calculated from the input SMILES (Simplified Molecular Input Line Entry System) string, (iii) the AD making it suitable for both well‐known small molecules as well as novel chemical entities.

Moreover, d4p_v1 also generates a visual representation of the 2D structure of the query molecule, allowing researchers to inspect the structure directly within the Colab notebook. This freely available tool lowers the barrier for researchers working in drug discovery and DPP‐4‐related research, accelerating the identification of promising drug candidates in the field of diabetes therapeutics.

## 3 Conclusion

The combination of fingerprint‐based QSAR models with theoretical calculations of electronic properties presents a valuable approach for exploring the structural needs of DPP‐4 inhibitors. These compounds are significant in addressing T2DM, inflammation, oxidative stress, appetite regulation, and various other health issues. Hence, DPP‐4 inhibitors are crucial tools in the fight against multiple diseases. In this study, the unique chemical structures of DPP‐4i influencing the biological activity are identified by the good and poor fingerprints derived from the Bayesian model. Structural fingerprints such as 4‐aminopiperidine groups are associated with reduced activity, suggesting that bulkier polar groups in certain regions may interfere with optimal binding. In contrast, 2‐cyanopyrrolidine, 3‐amino tetrahydropyran, and difluorophenyl groups are found to significantly enhance potency, reinforcing the importance of small polar and hydrophobic substituents. The 2‐cyanopyrrolidine (fingerprints G1, G5) containing compounds D0012 and D0013, exhibited a balanced profile, with their stability and reactivity parameters falling between those of D0010 (containing 3‐amino tetrahydropyran fingerprint G10) and D0094 (containing difluorophenyl fingerprint G14). This balance suggests that D0012 and D0013 may offer a favorable compromise between reactivity and stability, making them potentially versatile candidates for further exploration. In addition, the “d4p_v1,” an online cheminformatics tool, was developed by using a pre‐trained and thoroughly validated AI/ML model. It enables interested users to efficiently screen potential DPP‐4i. This open‐access tool accelerates DPP‐4 drug discovery by simplifying the screening process for potential inhibitors.

## 4 Experimental

### 4.1 Data Preparation

Biological activity data for DPP‐4 inhibitors with IC_50_ values were sourced from the BindingDB [38, 39]. After removing duplicates, entries lacking IC_50_ values, compounds with ranged values and compounds without simplified molecular input line entry system (SMILES) annotations were eliminated. Finally, the data set was refined to 3061 compounds.

### 4.2 Fingerprint‐Based Study

#### 4.2.1 Chemical Space Analysis

The relationships between the investigated DPP‐4i were explored through analysis using RDKit fingerprints [40]. The similarity between compounds was measured using the Tanimoto coefficient (*Tc*) [13, 14]. Bemis–Murcko scaffold analysis [17] was conducted to identify key molecular scaffolds of DPP‐4 inhibitors. The scaffold analysis was performed by Extended Connectivity Fingerprint (*ECFP4*) fingerprints [41] with the aid of Fasda_v1 tool (https://fasdav1web.streamlit.app/).

#### 4.2.2 Bayesian Classification Study

The Bayesian classification model was developed using Discovery Studio 3.0 (DS) software [42], employing the “*Create Bayesian Model*” protocol. The models were developed on the fingerprint descriptor *ECFP6* along with MW, *nHBD*, *nHBA*, *nRB*, *nAR*, lipophilicity (*AlogP*), and molecular fractional polar surface area (*MFPSA*). The model underwent the statistical validation procedures as described earlier [43].

### 4.3 Frontier Molecular Orbital Analysis and ESP Map

The DFT studies over the selected compounds D0010, D0012, D0013, D0094, along with the reference, were performed using the Gaussian 09 program [44]. To conduct the DFT studies, a hybrid functional B3LYP (Becke three‐parameter Lee–Yang–Parr) [45] was employed along with a split‐valence atomic basis set 6‐31+g(d) [46]. To check the minima of the compounds, frequency calculations were performed on the optimized geometries of the compounds. The absence of the imaginary frequency confirms the formation of the optimized geometries. The frontier molecular orbitals (FMO), that is, HOMO and LUMO were constructed to analyze the HOMO and LUMO distributions. The ESP maps were generated to visualize the molecular electron density and polarity of the compounds, which indicate the possible regions for the polar interactions. With the help of ESP plots, we can interpret the electron‐deficient and electron‐rich regions of the molecule, which are considered important to understand the interaction of the molecule with the protein. Chemical properties such as electrophilicity index (ω), chemical potential (µ), electronegativity (χ), hardness (ɳ), softness (S) were calculated using the following mathematical expressions [47]:

$$\omega =\frac{{µ}^{2}}{2ɳ},$$

$$µ=\frac{\left(E_{\mathrm{HOMO}}+E_{\mathrm{LUMO}}\right)}{2},$$

$$ɳ=\frac{E_{\text{LUMO}}-E_{\text{HOMO}}}{2},$$

$$S=\frac{1}{ɳ},$$

$$\chi =\frac{{-(E}_{\mathrm{HOMO}}+E_{\mathrm{LUMO}})}{2},$$

### 4.4 Machine Learning (ML) Study

#### 4.4.1 Descriptor Calculation and Feature Selection

Descriptors were computed using an *in‐house* Python script [29], which utilized the Mordred library [30] to calculate 1614 descriptors (except the 3D features). Descriptors with missing values, non‐numeric values, or those showing little to no variance were systematically removed from further analysis. Next, descriptor selection was carried out using the Information Gain (Mutual Information, MI) approach, retaining feature with importance scores above 0.15. While this method effectively reduces dimensionality, it does not account for interactions between descriptors and may overlook subtle but relevant features. To mitigate this, we employed robust ML algorithms (e.g., RF) capable of modeling descriptor interdependencies, and validated our models with cross‐validation to minimize potential bias from feature reduction.

#### 4.4.2 RF Model Development

The ML model was built using the RF algorithm, known for its flexibility and strong performance across diverse datasets [33]. Here, the hyperparameters of the RF model such as *n_estimators* (number of trees in the forest), *max_depth* (the maximum depth of each tree), *min_samples_split* (the minimum number of samples required to split an internal node), and *min_samples_leaf* (the minimum number of samples that a leaf node must have) were optimized by using *Optuna* [34, 35, 48]. The final models were evaluated using statistical metrics, as previously discussed [43].

## Ethics Statement

The authors have nothing to report.

## Consent

The authors have nothing to report.

## Conflicts of Interest

The authors declare no conflicts of interest.

## Code Availability

https://github.com/Amincheminfom/d4p_v1.

## Supporting information

Supplementary Materials.

## Data Availability

The data associated with this study are contained within the article or in the supporting material. If required, data will be made available on request.
