# Supplementary material for: AI/ML‐Driven DPP‐4 Inhibitor Predictor (d4p_v1) for Enhanced Type 2 Diabetes Mellitus Management: Insights Into Chemical Space, Fingerprints, and Electrostatic Potential Maps
Source: Arch Pharm (Weinheim). 2025 Sep 22;358(9):e70106. doi: 10.1002/ardp.70106 (PMC12455174; doi:10.1002/ardp.70106)
Supplement: Supplementary file 1 — Supplementary Materials. [file ARDP-358-e70106-s001.doc]

**Supplementary Files**

**AI/ML-driven DPP-4 inhibitor predictor (d4p_v1) for enhanced type 2 diabetes mellitus management: Insights into chemical space, fingerprints and electrostatic potential maps**

Anu Manhas^1^,*, Ritam Dutta^2^, Stefano Piotto^3^, Sk. Abdul Amin^3^,*

^1^*Department of Chemistry, School of Energy Technology, Pandit Deendayal Energy University, 382426, India.*

*^2^Department of Pharmaceutical Technology, JIS University, Kolkata, West Bengal 700108, India.*

*^3^Department of Pharmacy, University of Salerno, Via Giovanni Paolo II 132, 84084 Fisciano, SA, Italy.*

*Corresponding authors:

Dr. Anu Manhas (Orcid ID: [0000-0003-2759-9845](https://orcid.org/0000-0003-2759-9845))

Email: [Anu.Manhas@sot.pdpu.ac.in](mailto:Anu.Manhas@sot.pdpu.ac.in)

Dr. Sk. Abdul Amin (Orcid ID: [0000-0003-4799-7322](https://orcid.org/0000-0003-4799-7322))

Email: [pharmacist.amin@gmail.com](mailto:pharmacist.amin@gmail.com) & [askabdul@unisa.it](mailto:askabdul@unisa.it)


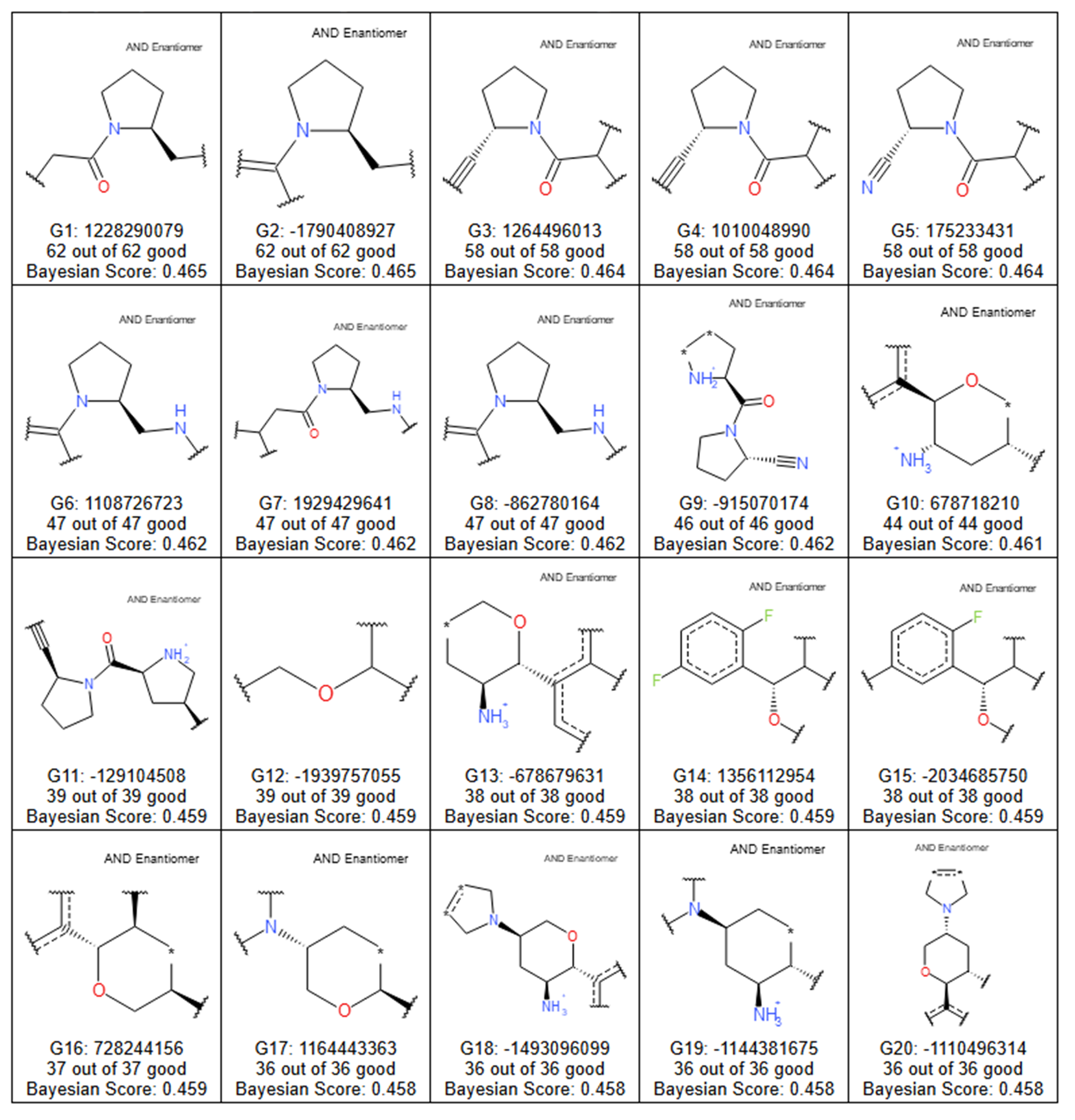


**Figure S1.** Bayesian good fingerprint (G1-G20) that positively influence DPP-4 inhibitory activity.


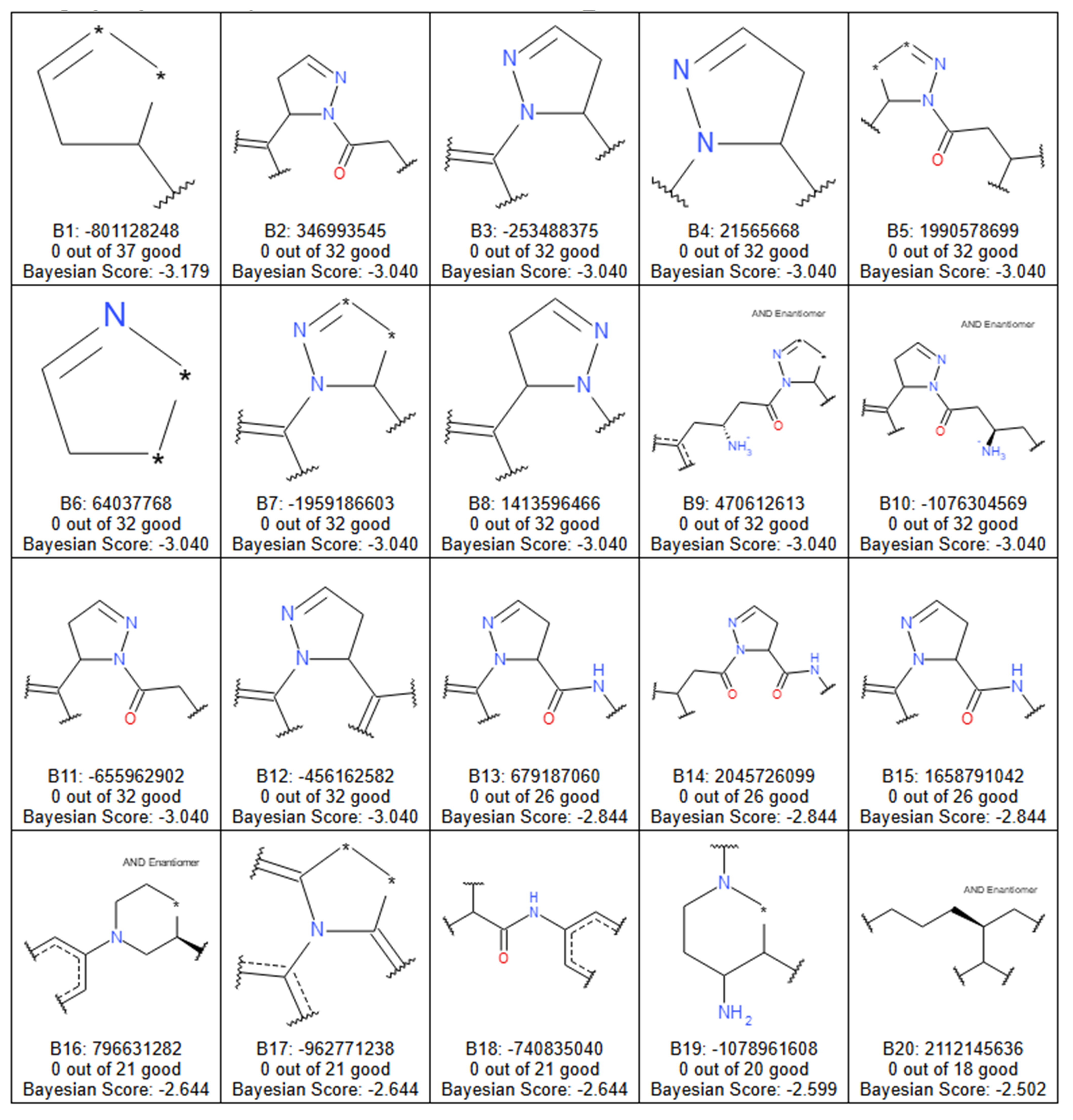


**Figure S2.** Bayesian bad fingerprint (B1-B20) that negatively influence DPP-4 inhibitory activity.


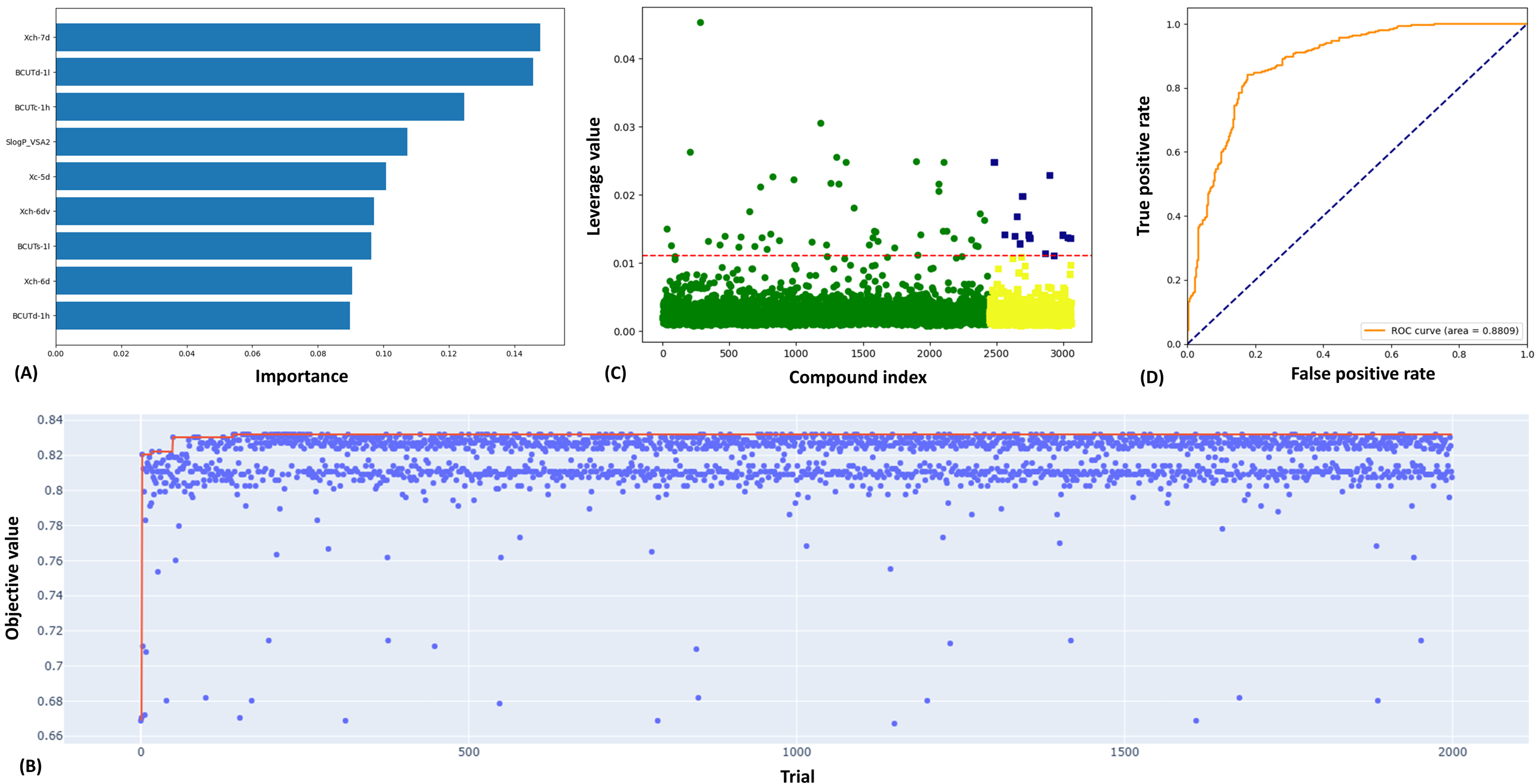


**Figure S3**. (A) Feature importance plot, (B) Hyperparameter optimization process of finding the optimal values for *max_depth*, *min_samples_leaf*, *min_samples_split*, *n_estimators*. 2000 runs have been performed to find the optimal values, (C) Plot of the applicability domain, (D) ROC plot of the developed model.
